# Docking domains from modular polyketide synthases and their use in engineering

**DOI:** 10.1038/s41467-025-61435-4

**Published:** 2025-07-22

**Authors:** Sabrina Collin, Kira J. Weissman

**Affiliations:** Université de Lorraine, CNRS, IMoPA, F-54000 Nancy, France

**Keywords:** Multienzyme complexes, Synthetic biology, Metabolic engineering, Natural product synthesis

**arising from** Y. Liu et al. *Nature Communications* 10.1038/s41467-025-55973-0 (2025)

In a recent paper entitled “Improving polyketide biosynthesis by rescuing the translation of truncated mRNAs into functional polyketide synthase subunits”, Liu et al.^1^ discovered that >93% of the mRNAs resulting from transcription of gigantic modular polyketide synthase (PKS) genes are abridged, leading to reduced overall concentrations of complete assembly lines, and thus biosynthetic product. Although the authors’ strategy of splitting the genes into smaller fragments by introducing docking domains (DDs) did not reduce the number of truncated transcripts, it did increase the overall proportion and rate of translation of intact modules farther down the PKSs, resulting in a 5−13-fold boost in metabolite titers. However, applying this approach to additional PKS systems will depend on information that was almost entirely absent from the published manuscript. While our critique does not affect the central conclusions of the paper^1^, we provide a detailed analysis of the employed DDs that suggests that the engineering might have resulted in side products, and propose succinct guidelines for future docking domain-based manipulation of modular PKSs.

Bacteria harbor gigantic molecular-scale assembly lines called modular polyketide synthases (PKSs)^2^ that build polyketide specialized metabolites. Each PKS multienzyme contains one or multiple modules, where each module is responsible for growing the polyketide chain by one building block and its chemical modification. As polyketides have found extensive use in human and veterinary medicine, there is substantial interest in enhancing their yields. In this context, Liu and colleagues’ finding that mRNAs encoding large PKS subunits are truncated is important, as it implies that smaller genes are better. Supporting this idea, inspection of all of the *cis*-acyltransferase (AT) PKSs in the MIBiG^3^ database – a curated collection of experimentally characterized systems – shows that 88% of the 462 catalogued subunits comprise 3 or fewer modules, and fully 72% have 1 or 2 (Fig. 1 and Supplementary Data 1).

**Fig. 1:**
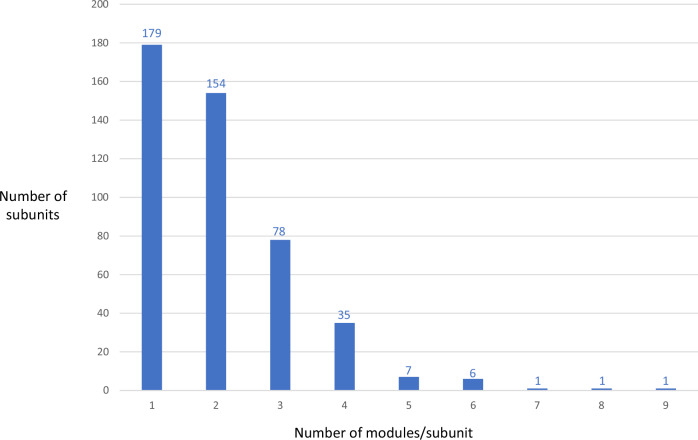
Composition in modules of the *cis*-AT PKS subunits catalogued in the MIBiG database^3^. This analysis reveals a strong preference for polypeptides incorporating three or fewer modules. The effect on polyketide yields identified by Liu et al.^1^ may explain this natural bias.

To separate the target PKSs into parts, Liu et al. replaced the short linker regions which covalently join modules together within subunits, with pairs of docking domains^4^— a strategy first reported in 2009^5^, although this seminal work was not cited in the manuscript. DDs are protein-protein recognition motifs situated at the extreme C- and N-terminal ends of native PKS multienzymes. Via highly specific, non-covalent contacts, they ensure that the PKS polypeptides line up in the defined order that is critical to maintaining product fidelity. It is therefore not the case as stated by the authors that “…docking domains do not enhance the communication and activity of modular PKS”, as it has been shown by both deletion and swapping of these regions that they contribute to biosynthetic efficacy^6^.

Based on several decades of research carried out in our and other laboratories, we now have a good understanding of both DD structure and function. Globally, this work has revealed that the *cis*-AT class of modular PKSs deploys at least five different types of DD, as judged by the three-dimensional structures of the docked complexes (referred to as 1a^4^, 1b^7^ and 2^8^ (Fig. 2a−c), as well as 1-like^9^ and 2-like^9^). In each case, both the C-terminal DD (^C^DD) and the N-terminal DD (^N^DD) are composed of α-helices, but of variable number and amino acid composition, leading to overall DD assemblies of distinct topology (Fig. 2a−c). The high specificity of interaction within the DD types arises from strategically positioned electrostatic interactions that drive association^4,7,8^, as well as from shape complementarity, while the different DD types are intrinsically orthogonal to each other. Thus, in PKS systems, the same type of DD may operate at multiple intersubunit junctions, but equally, several types of DD (e.g. both 1a and 1b) may be present^9^.

**Fig. 2:**
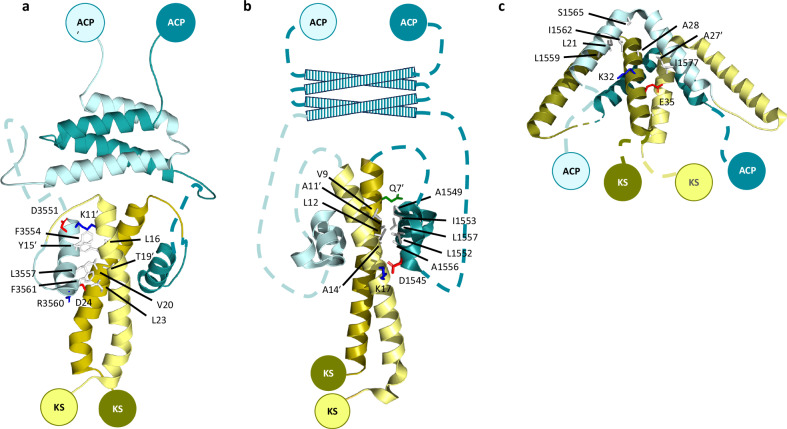
Structures of complexes of three of the identified types of docking domains from *cis*-AT PKS systems. **a** Type 1a^4^. **b** Type 1b^7^. **c** Type 2^8^. The C-terminal docking domains are indicated in shades of blue, and the N-terminal docking domains in shades of gold. For each docking complex, the residues comprising one of the interfaces are shown with the following color code: red: negatively charged; blue: positively charged; green: polar; white: hydrophobic. The primes denote residues contributed by the second monomer. (Note: the dashed lines indicate regions whose structures have not been characterized). The structures in this figure were produced using PYMOL^15^.

Based on this information, it is evident that the docking domains used to split PKS multienzymes into shorter proteins should not interact with those native to the system. In this way, the engineering will preserve the normal ordering of the subunits/modules. Conversely, if aberrant cross-talk occurs between the inserted and natural DDs, the subunits may assume non-native configurations. The result may be alternative products or stalled assembly lines, both of which would reduce yields of the target product. The manuscript by Li et al. lacks any discussion of the compatibility of the transplanted DDs with the recipient PKSs, and in particular, their potential to engage in mis-associations. We therefore carried out a comprehensive analysis of docking in the three PKSs investigated by the authors (butenyl-spinosyn (Bus), avermectin (AveA) and epothilone (Epo)), as well as of the three pairs of inserted DDs. For this, the sequences of the putative docking regions were compared to representatives of the 1a, 1b and 2 DD types^9^ (Supplementary Figs. 1−3).

This analysis revealed that the butenyl-spinosyn PKS incorporates 3 type 1a DD pairs (at the BusB/BusC, BusC/BusD and BusD/BusE interfaces), and 1 set of type 1b DDs (BusA/BusB junction) (Supplementary Figs. 1, 2). The salinomycin (Sln) DDs (SlnA1 ^C^DD/SlnA2 ^N^DD and SlnA7 ^C^DD/SlnA8 ^N^DD) that were used to split the trimodular BusA into two or three fragments are both type 1a (Supplementary Fig. 1). This choice, although motivated by the absence of terminators in the respective *sln* genes, set the bar high in terms of specificity. Indeed, the basic mode of docking in the engineered PKSs is the same at four or five intersubunit junctions, meaning that partner choice is entirely dependent on a limited set of interface residues. More detailed inspection of the twelve charged and hydrophobic residues that make up the interface in each pair^4^ suggests that both SlnA1 ^C^DD and SlnA7 ^C^DD could be compatible with BusD ^N^DD, and reciprocally, that the BusC ^C^DD could interact productively with either SlnA1 ^N^DD or SlnA8 ^N^DD (Table 1). No attempt was made by the authors to search for side products potentially arising from these incorrect associations, and thus it cannot be excluded that the yield increases would have been even greater if alternative DD pairs were employed.

**Table 1:**
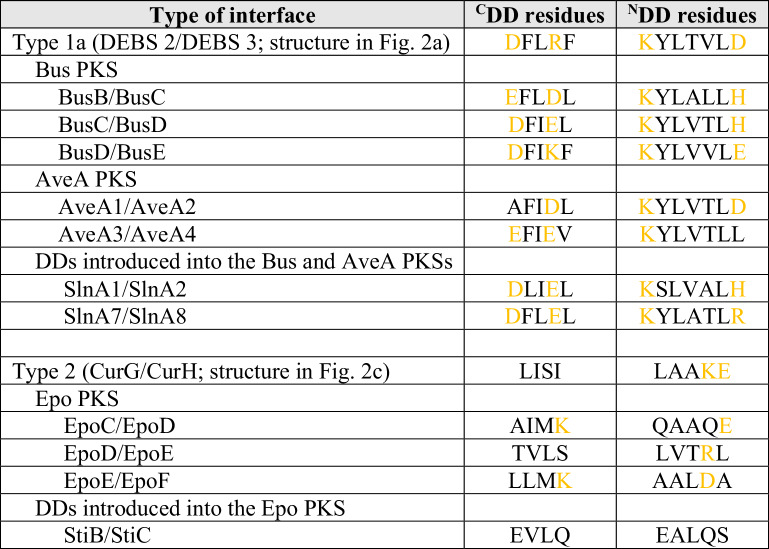
Comparison of the ensemble of interface residues in the systems that were engineered, relative to the introduced pairs of docking domains^a^

^a^The interface positions in which charged residues contribute to complex formation are indicated in gold (as in Supplementary Fig. 1−3).

In the case of the avermectin system, two type 1a and 1 type 1b junctions are present (Supplementary Figs. 1, 2). The differences between the native AveA and the introduced type 1a Sln DD pairs are more substantial across the key residues (Table 1), but again, no direct evidence was provided that orthogonality, and thus the native sequence of modules, was maintained. Concerning the Epo system, it is a hybrid between four *cis*-AT PKS subunits (EpoA, C−F) and a single subunit of nonribosomal peptide synthetase (NRPS) (EpoB). The PKS/NRPS and NRPS/PKS docking elements are distinct from the five types typical of *cis*-AT PKSs (e.g. a SLIM/β-hairpin pair operates at the EpoA/EpoB interface^10,11^), while those at the remaining PKS/PKS interfaces are type 2 (Supplementary Fig. 3). In this case, the authors elected to use an additional pair of type 2 domains from the stigmatellin PKS (StiB ^C^DD/StiC ^N^DD, Supplementary Fig. 3) to split the tetramodular EpoD into two bimodular proteins. Again, the result of this choice was to increase to four the number of junctions in the resulting PKS dependent on the same type of docking domain, necessitating direct verification that improper interactions do not occur.

The failure to address specificity issues is surprising, as it is already possible based on existing literature to define guidelines for docking domain-based engineering that ensure that orthogonality is preserved. These include: (i) inserting a type of DD which is not already present in the system; (ii) using a non-natural (e.g. synthetic) pair of DDs, such as the SYNZIPs that have been shown to function in engineered PKSs^12^; and (iii) replacing all sets of DDs within the engineered PKS with the native pairs of DDs from a system incorporating at least as many interfaces (e.g. the stambomycin PKS, which comprises 6 pairs of type 1a DDs and 2 pairs of type 1b DDs^13^). We do not recommend attempting to modify partner choice within DD types by site-directed mutagenesis, as it is not yet possible to reliably predict docking specificity. Ultimately, direct confirmation of the intrinsic orthogonality of all DD sets can be obtained via biophysical analysis of recombinant or synthetic DD peptides, as previously described^9,13^.

## Methods

### Analysis of subunit composition and docking domains

The subunits used for the analysis of modular composition (Fig. 1 and Supplementary Data 1) were sourced from the MIBiG database (https://mibig.secondarymetabolites.org/)^3^. The sequences of representative type 1a, type 1b and type 2 ^C^DDs and ^N^DDs used in Fig. 2, Table 1, and Supplementary Figs. 1−3 were obtained from previously published analyses^9,10^. To identify the ^C^DDs in the Bus, AveA and Epo systems which had not been analyzed earlier, the C-terminal boundaries of the upstream domains (ACP or peptidyl carrier protein (PCP)) were determined by multiple sequence alignment of the subunit ends using ClustalOmega (https://www.ebi.ac.uk/jdispatcher/msa/clustalo)^14^, with the remaining region considered as ^C^DD. Similarly, the extent of the ^N^DDs was determined by alignment of the first portion of the corresponding subunits, which allowed identification of the start sites of the KS domains downstream of the ^N^DDs. Supplementary Figs. 1−3 were generated using ClustalOmega^14^.

### Reporting summary

Further information on research design is available in the Nature Portfolio Reporting Summary linked to this article.

## Supplementary information

Supplementary Information

Description of Additional Supplementary Files

Supplementary Data 1

Reporting Summary

## Data Availability

Data supporting the findings of this work are available within the paper and its Supplementary Information files. A reporting summary for this Article is available as a Supplementary Information file.
