# Supplementary material for: Docking domains from modular polyketide synthases and their use in engineering
Source: Nat Commun. 2025 Jul 22;16:6690. doi: 10.1038/s41467-025-61435-4 (PMC12284057; doi:10.1038/s41467-025-61435-4)
Supplement: Supplementary file 1 — Supplementary Information [file 41467_2025_61435_MOESM1_ESM.pdf]

Docking domains from modular polyketide synthases and their use in engineering

Collin and Weissman

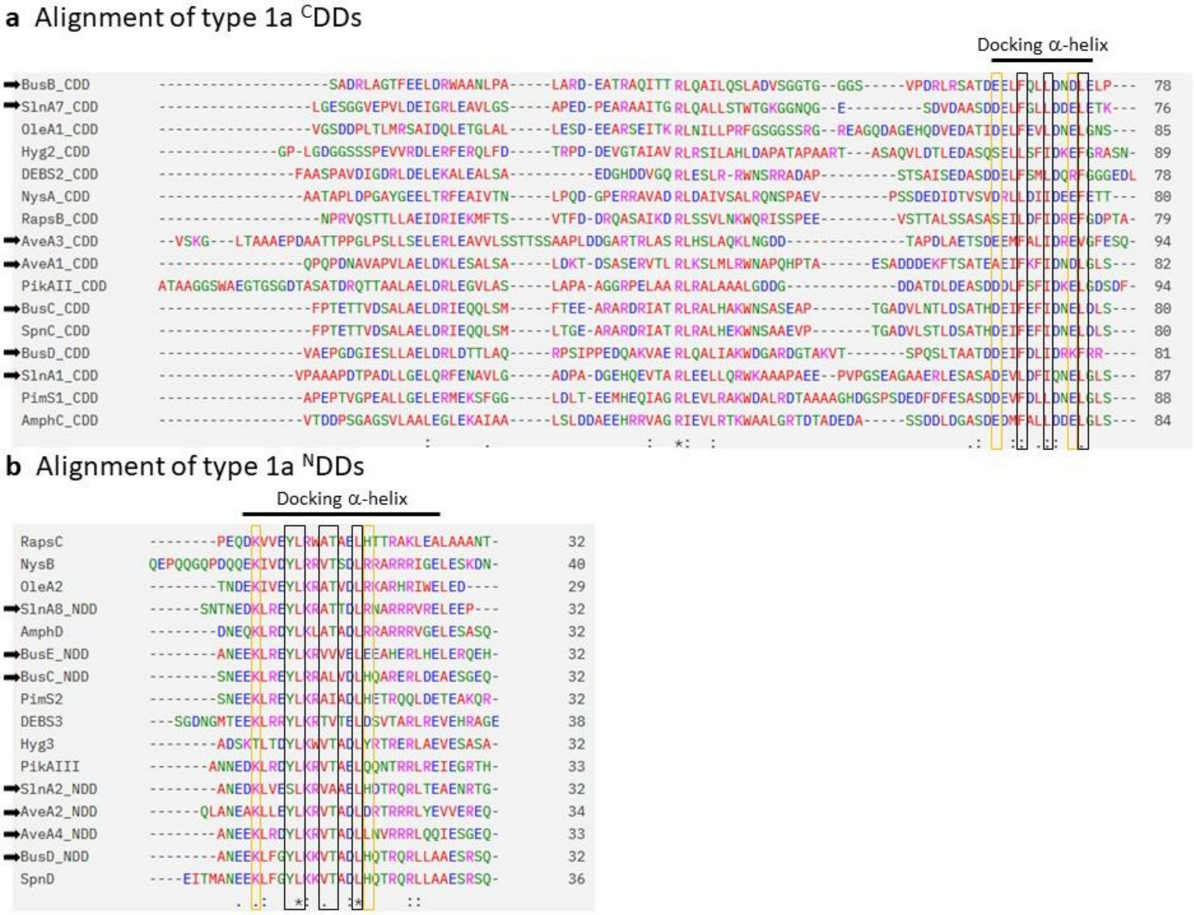

Supplementary Fig. 1. Multiple sequence alignment of pairs of type 1a docking domains[1]. **a** Alignment of type 1a ^{C}DDs. **b** Alignment of type 1a ^{N}DDs. The residues comprising the docking α-helices and the interface are based on PDB IDs 1PZQ and 1PZR, respectively, with hydrophobic residues indicated by black boxes and charged residues, by yellow boxes. The DD pairs directly relevant to this article are indicated with arrows. Key to PKS systems: Bus, butenyl-spinosyn[2]; SlnA, salinomycin[3]; OleA, oleandomycin[4]; Hyg, hygromycin[5]; DEBS, 6-deoxyerythronolide B[6]; Nys, nystatin[7]; Raps, rapamycin[8]; AveA, avermectin[9]; PikA, pikromycin[10]; Spn, spinosad[11]; PimS, pimaricin[12]; Amph, amphotericin[13]. The subunit nomenclature indicates the order of multienzymes within each system.

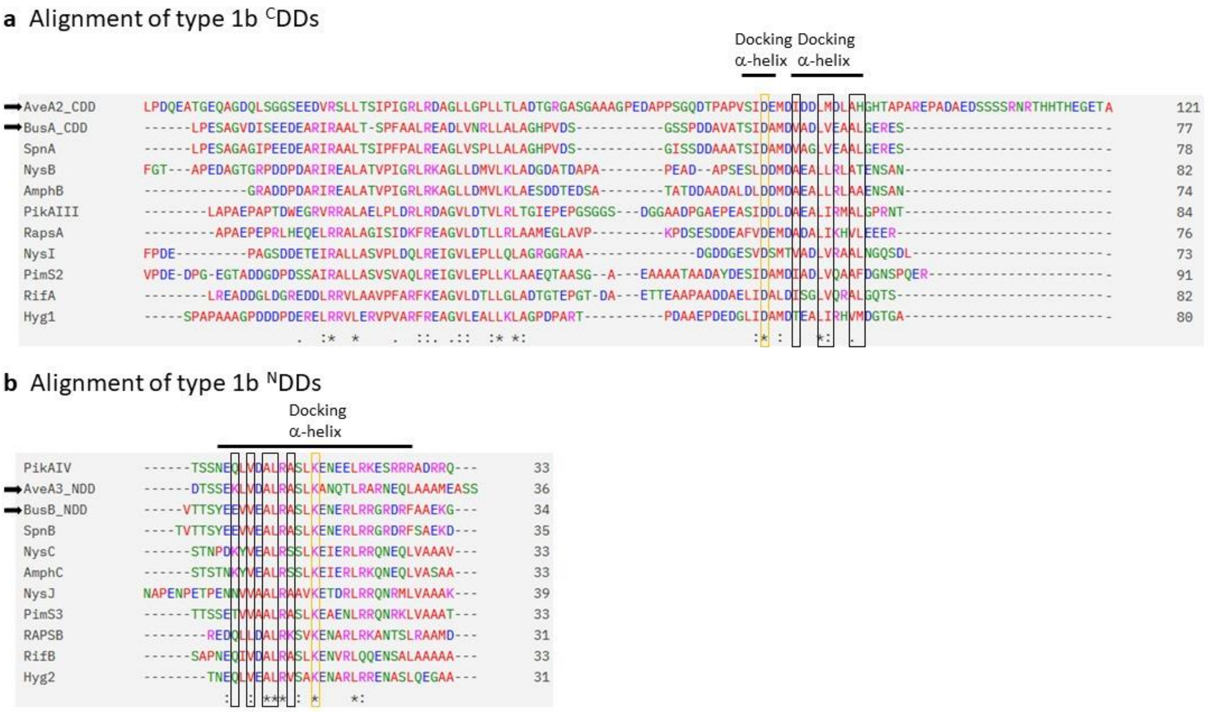

Supplementary Fig. 2. Multiple sequence alignment of pairs of type 1b docking domains[14]. **a** Alignment of type 1b ^{C}DDs. **b** Alignment of type 1b ^{N}DDs. The residues comprising the docking α-helices and the interface are based on PDB 3F5H, with hydrophobic residues indicated by black boxes and charged residues, by yellow boxes. The DD pairs directly relevant to this article are indicated with arrows. Key to PKS systems: AveA, avermectin[9]; Bus, butenyl-spinosyn[2]; Spn, spinosad[11]; Nys, nystatin[7]; Amph, amphotericin[13]; Rif, rifamycin[15,16]; Hyg, hygromycin[5]; Raps, rapamycin[8]; PikA, pikromycin[10]; Nys, nystatin[7]; PimS, pimaricin[12]. The subunit nomenclature indicates the order of multienzymes within each system.

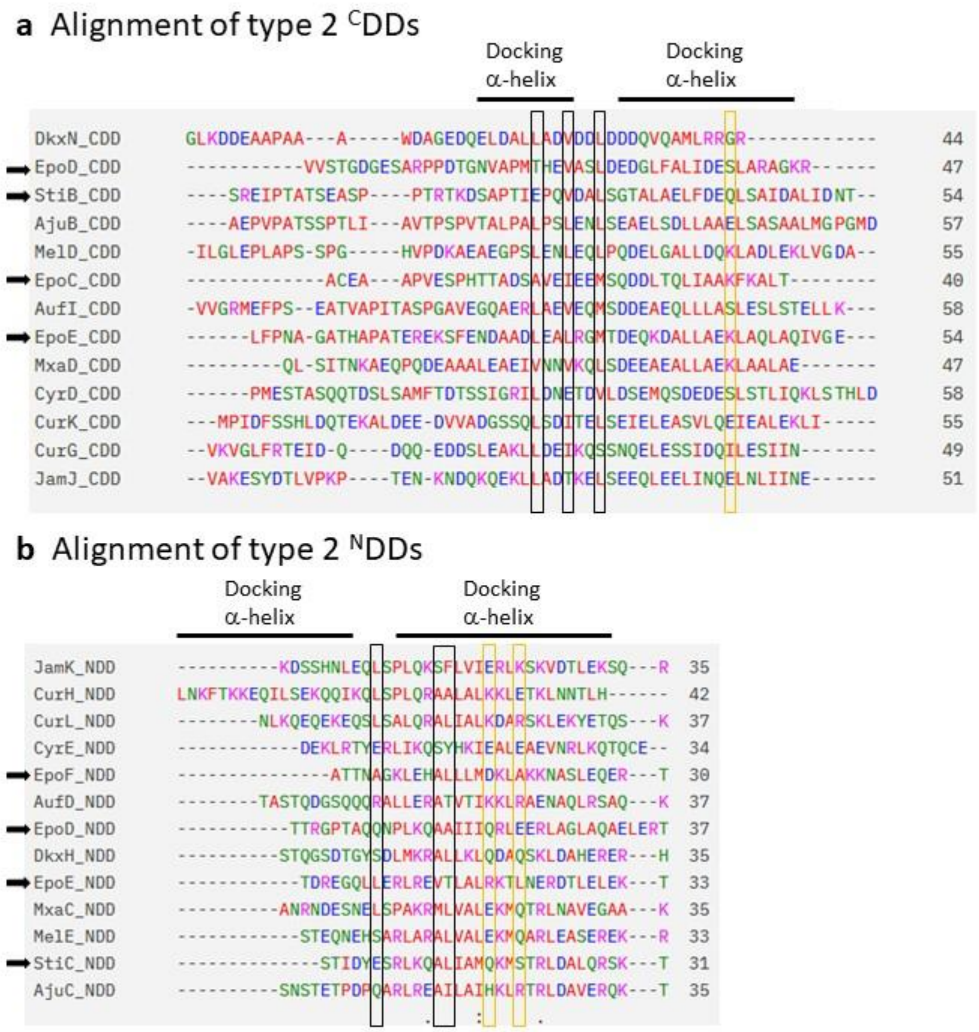

Supplementary Fig. 3. Multiple sequence alignment of pairs of type 2 docking domains[17]. **a** Alignment of type 2 ^{C}DDs. **b** Alignment of type 2 ^{N}DDs. The residues comprising the docking α-helices and the interface are based on PDB 4MYY, with hydrophobic residues indicated by black boxes and charged residues, by yellow boxes. The DD pairs directly relevant to this article are indicated with arrows. Key to PKS systems: Dkn, DKxanthene[18]; Epo, epothilone[19]; Sti, stigmatellin[20]; Aju, ajudazol[21]; Mel, melithiazol[22]; Auf, aurofurone[23]; Mxa, myxalamid[24]; Cyr, cylindrospermopsin[25]; Cur, curacin[26]; Jam, jamaicamide[27]. The subunit nomenclature indicates the order of multienzymes within each system.

Supplementary references

1. Broadhurst, R. W., Nietlispach, D., Wheatcroft, M. P., Leadlay, P. F. & Weissman, K. J. The structure of docking domains in modular polyketide synthases. *Chem. Biol.* **10**, 723–731 (2003).
2. Zaburannyi, N., Rabyk, M., Ostash, B., Fedorenko, V. & Luzhetskyy, A. Insights into naturally minimised *Streptomyces albus* J1074 genome. *BMC Genomics* **15**, 97 (2014).
3. Jiang, C., Wang, H., Kang, Q., Liu, J. & Bai, L. Cloning and characterization of the polyether salinomycin biosynthesis gene cluster of *Streptomyces albus* XM211. *Appl. Environ. Microbiol.* **78**, 994–1003 (2012).
4. Shah, S. *et al.* Cloning, characterization and heterologous expression of a polyketide synthase and P-450 oxidase involved in the biosynthesis of the antibiotic oleandomycin. *J. Antibiot.* **53**, 502–508 (2000).
5. Ruan, X., Stassi, D., Lax, S. A. & Katz, L. A second type-I PKS gene cluster isolated from *Streptomyces hygroscopicus* ATCC 29253, a rapamycin-producing strain. *Gene* **203**, 1–9 (1997).
6. Cortés, J., Haydock, S. F., Roberts, G. A., Bevitt, D. J. & Leadlay, P. F. An unusually large multifunctional polypeptide in the erythromycin-producing polyketide synthase of *Saccharopolyspora erythraea*. *Nature* **348**, 176–178 (1990).
7. Brautaset, T. *et al.* Biosynthesis of the polyene antifungal antibiotic nystatin in *Streptomyces noursei* ATCC 11455: analysis of the gene cluster and deduction of the biosynthetic pathway. *Chem. Biol.* **7**, 395–403 (2000).
8. Aparicio, J. F. *et al.* Organization of the biosynthetic gene cluster for rapamycin in *Streptomyces hygroscopicus*: analysis of the enzymatic domains in the modular polyketide synthase. *Gene* **169**, 9–16 (1996).
9. Ikeda, H., Nonomiya, T., Usami, M., Ohta, T. & Omura, S. Organization of the biosynthetic gene cluster for the polyketide anthelmintic macrolide avermectin in *Streptomyces avermitilis*. *Proc. Natl. Acad. Sci. U.S.A.* **96**, 9509–9514 (1999).
10. Xue, Y., Wilson, D. & Sherman, D. H. Genetic architecture of the polyketide synthases for methymycin and pikromycin series macrolides. *Gene* **245**, 203–211 (2000).
11. Waldron, C. *et al.* Cloning and analysis of the spinosad biosynthetic gene cluster of *Saccharopolyspora spinosa*. *Chem. Biol.* **8**, 487–499 (2001).
12. Aparicio, J. F., Fouces, R., Mendes, M. V., Olivera, N. & Martín, J. F. A complex multienzyme system encoded by five polyketide synthase genes is involved in the

biosynthesis of the 26-membered polyene macrolide pimaricin in *Streptomyces natalensis*. *Chem. Biol.* **7**, 895–905 (2000).

13. Caffrey, P., Lynch, S., Flood, E., Finnan, S. & Oliynyk, M. Amphotericin biosynthesis in *Streptomyces nodosus*: deductions from analysis of polyketide synthase and late genes. *Chem. Biol.* **8**, 713–723 (2001).
14. Buchholz, T. J. *et al.* Structural basis for binding specificity between subclasses of modular polyketide synthase docking domains. *ACS Chem. Biol.* **4**, 41–52 (2009).
15. Schupp, T., Toupet, C., Engel, N. & Goff, S. Cloning and sequence analysis of the putative rifamycin polyketide synthase gene cluster from *Amycolatopsis mediterranei*. *FEMS Microbiol Lett.* **159**, 201–207 (1998).
16. August, P. R. *et al.* Biosynthesis of the ansamycin antibiotic rifamycin: deductions from the molecular analysis of the *rif* biosynthetic gene cluster of *Amycolatopsis mediterranei* S699. *Chem. Biol.* **5**, 69–79 (1998).
17. Whicher, J. R. *et al.* Cyanobacterial polyketide synthase docking domains: a tool for engineering natural product biosynthesis. *Chem. Biol.* **20**, 1340–1351 (2013).
18. Meiser, P. *et al.* DKxanthene biosynthesis – understanding the basis for diversity-oriented synthesis in myxobacterial secondary metabolism. *Chem. Biol.* **15**, 771–781 (2008).
19. Tang, L. *et al.* Cloning and heterologous expression of the epothilone gene cluster. *Science* **287**, 640–642 (2000).
20. Gaitatzis, N. *et al.* The biosynthesis of the aromatic myxobacterial electron transport inhibitor stigmatellin is directed by a novel type of modular polyketide synthase. *J. Biol. Chem.* **277**, 13082–13090 (2002).
21. Buntin, K. *et al.* Production of the antifungal isochromanone ajudazols A and B in *Chondromyces crocatus* Cm c5: biosynthetic machinery and cytochrome P450 modifications. *Angew. Chem. Int. Ed. Engl.* **47**, 4595–4599 (2008).
22. Weinig, S., Hecht, H.-J., Mahmud, T. & Müller, R. Melithiazol biosynthesis: further insights into myxobacterial PKS/NRPS systems and evidence for a new subclass of methyl transferases. *Chem. Biol.* **10**, 939–952 (2003).
23. Frank, B. *et al.* From genetic diversity to metabolic unity: studies on the biosynthesis of aurafurones and aurafuron-like structures in myxobacteria and streptomycetes. *J. Mol. Biol.* **374**, 24–38 (2007).
24. Silakowski, B., Nordsiek, G., Kunze, B., Blöcker, H. & Müller, R. Novel features in a combined polyketide synthase/non-ribosomal peptide synthetase: the myxalamid

biosynthetic gene cluster of the myxobacterium *Stigmatella aurantiaca* Sga15. *Chem Biol* **8**, 59–69 (2001).

25. Jiang, Y. *et al.* Sporadic distribution and distinctive variations of cylindrospermopsin genes in cyanobacterial strains and environmental samples from Chinese freshwater bodies. *Appl. Environ. Microbiol.* **80**, 5219–5230 (2014).
26. Chang, Z. *et al.* Biosynthetic pathway and gene cluster analysis of curacin A, an antitubulin natural product from the tropical marine cyanobacterium *Lyngbya majuscula*. *J. Nat. Prod.* **67**, 1356–1367 (2004).
27. Edwards, D. J. *et al.* Structure and biosynthesis of the jamaicamides, new mixed polyketide-peptide neurotoxins from the marine cyanobacterium *Lyngbya majuscula*. *Chem. Biol.* **11**, 817–833 (2004).
