# Supplementary material for: Docking domains from modular polyketide synthases and their use in engineering
Source: Nat Commun. 2025 Jul 22;16:6690. doi: 10.1038/s41467-025-61435-4 (PMC12284057; doi:10.1038/s41467-025-61435-4)
Supplement: Supplementary file 2 — Description of Additional Supplementary Files [file 41467_2025_61435_MOESM2_ESM.docx]

**Description of Additional Supplementary Files**

File Name: Supplementary Data 1

Description: Number of modules/subunit in the *cis*-AT PKS systems catalogued in the MIBiG database (<https://mibig.secondarymetabolites.org/repository)1>.

**Reference**

Zdouc, M. M. *et al.* MIBiG 4.0: advancing biosynthetic gene cluster curation through global collaboration. *Nucleic Acids Res.* **53**, D678–D690 (2025).
